# Update on the comparative *in vitro* activity of cefiderocol and four β-lactam–β-lactamase-inhibitor combinations against clinically important Gram-negative pathogens

**DOI:** 10.1093/jacamr/dlag103

**Published:** 2026-06-08

**Authors:** E Wohlfarth, F Deuchert, S G Gatermann, P G Higgins, Y Pfeifer, N Pfennigwerth, H Seifert, G Werner, Study Group ‘Antimicrobial Resistance’ of the Paul-Ehrlich-Society for Infection Therapy, Sören Becker, Achim Hörauf, Gunnar Hischebeth, Martin Holfelder, Ulrich Eigner, Sören Gatermann, Hannes Stapmanns, Andreas Diefenbach, Andreas Knaust, Eberhard Kniehl, Andrea Becker, Karsten Becker, Jürgen Bohnert, Jürgen Rödel, Bettina Löffler, Dietrich Mack, Mathias Kolbert, Colin MacKenzie, Can Imirzalioglu, Alexander Mellmann, Evgeny Idelevich, Andreas Podbielski, Anette Devid, Peter-Michael Rath, Ulrike Scharrmann, Jonathan Jantsch, André Gessner, Sabine Schubert, Sören Schubert, Carolina Mattwich, Ulrike Schumacher, Harald Seifert, Danuta Stefanik, Ekkehard Siegel, Nadja Joss, Heike Weißer, Bettina Herbach, Thomas Wichelhaus, Stefan Ziesing, Christiane Reineke

**Affiliations:** Antiinfectives Intelligence GmbH, Gottfried-Hagen-Str. 60-62, Cologne 51105, Germany; Antiinfectives Intelligence GmbH, Gottfried-Hagen-Str. 60-62, Cologne 51105, Germany; German National Reference Centre for Multidrug-Resistant Gram-Negative Bacteria, Ruhr-University Bochum, Universitätsstr. 150, Bochum 44801, Germany; Faculty of Medicine and University Hospital Cologne, Institute for Medical Microbiology, Immunology and Hygiene, Goldenfelsstr. 19-21, Cologne 50935, Germany; Institute of Translational Research, CECAD Cluster of Excellence, University of Cologne, Joseph-Stelzmann-Straße 26, Cologne 50931, Germany; German National Reference Centre for Multidrug-Resistant Gram-Negative Bacteria, Ruhr-University Bochum, Universitätsstr. 150, Bochum 44801, Germany; Faculty of Medicine and University Hospital Cologne, Institute for Medical Microbiology, Immunology and Hygiene, Goldenfelsstr. 19-21, Cologne 50935, Germany; Institute of Translational Research, CECAD Cluster of Excellence, University of Cologne, Joseph-Stelzmann-Straße 26, Cologne 50931, Germany; German Centre for Infection Research, partner Site Bonn-Cologne, Germany; Robert Koch-Institute, FG13 Nosocomial Pathogens and Antibiotic Resistances, Burgstr. 37, Wernigerode 38855, Germany

## Abstract

**Objectives:**

Antimicrobial resistance is considered one of the most dangerous threats to public health globally. In the last decade, several antimicrobial agents were developed for the treatment of infections with multidrug-resistant Gram-negative bacteria, especially carbapenem-resistant isolates. The aim of this study was to investigate the *in vitro* activity of the siderophore cephalosporin cefiderocol (FDC) and four comparator beta-lactam–beta-lactamase-inhibitor combinations (ceftazidime/avibactam, ceftolozane/tazobactam, imipenem/relebactam and meropenem/vaborbactam) against 401 clinical isolates of Gram-negative pathogens collected in Germany between 2019 and 2020.

**Methods:**

Genetic determinants of resistance were investigated by PCR and/or whole-genome sequencing. Furthermore, the ComASP test kit for cefiderocol minimum-inhibitory concentration (MIC) determination was compared to the EUCAST reference method broth microdilution.

**Results:**

The cefiderocol resistance rate of *Pseudomonas aeruginosa* was 1.5% while resistance to the comparator agents ranged from 19.7% to 40.9%. Among both *Acinetobacter baumannii* and *Stenotrophomonas maltophilia* isolates the MIC50 value of cefiderocol was six or more log_2_ steps lower than the ones of the comparator agents. Seven isolates (three *Escherichia coli*, one *Klebsiella pneumoniae*, one *P. aeruginosa* and two *A. baumannii*) revealed cefiderocol MICs > 2 mg/L. While different beta-lactamase genes were detected in these isolates, they were most probably not solely responsible for cefiderocol resistance. MICs produced by ComASP test kits were comparable to the results of broth microdilution in *E. coli*, *K. pneumoniae*, *P. aeruginosa* and *S. maltophilia* (categorical agreement > 95%; essential agreement > 91%).

**Conclusions:**

Overall, cefiderocol activity against *P. aeruginosa, A. baumannii* and *S. maltophilia* was higher compared to the other agents tested.

## Introduction

While the discovery of antibiotics has tremendously impacted modern medicine and extended the average life expectancy of humans by 23 years,^1^ their efficacy is under constant threat due to the spread of resistant pathogenic bacteria, both in the community and in health care facilities.^2^

The WHO has classified certain Gram-negative species with specific resistant phenotypes as ‘critical priority’ for the development of new antibiotics. This includes carbapenem-resistant Enterobacterales, third-generation cephalosporin-resistant Enterobacterales as well as carbapenem-resistant *Acinetobacter baumannii*. Meanwhile, carbapenem-resistant *Pseudomonas aeruginosa* has been reclassified into the second highest ‘high priority’ group as of 2024.^3^ The main mechanisms of resistance to third-generation cephalosporins, carbapenems and novel beta-lactamase inhibitors (BLIs) are production of extended-spectrum beta-lactamases (ESBL) and carbapenemases, respectively. ESBL and carbapenemase genes are often located on mobile genetic elements, such as plasmids, which enable the horizontal spread of these resistance determinants in various Gram-negative genera/species.^3^ Many of the newer antimicrobial agents rely on combining already established beta-lactams (BL) with BLI such as the cephalosporins ceftazidime with avibactam and ceftolozane with tazobactam or the carbapenems imipenem with relebactam and meropenem with vaborbactam. While these newer inhibitor agents are considered very effective against most Gram-negative bacteria, their activity often does not affect metallo-beta-lactamase (MBL) producers.^4^

Cefiderocol is a siderophore cephalosporin that forms chelating complexes with trivalent extracellular iron, enabling its active transport into bacterial cells.^5^ Together with the aforementioned BL/BLI combinations, cefiderocol is often considered a promising new option for the treatment of the Gram-negative pathogens of the ‘critical’ WHO classification.^6^ This is particularly important in this context due to its ability to withstand hydrolysis mediated by clinically relevant beta-lactamases such as MBLs or oxacillinase (OXA)-type carbapenemases.^7^

In two previous studies, we have demonstrated the activity of cefiderocol against clinically relevant Enterobacterales and nonfermenting Gram-negative isolates collected in 2013/14 and 2016/17, respectively. The activity of cefiderocol was compared to the four BL/BLI combinations ceftolozane/tazobactam, ceftazidime/avibactam, imipenem/relebactam and meropenem/vaborbactam, which generally showed less activity against *P. aeruginosa* and *S. maltophilia* isolates.^8,9^ The aim of the present study was to compare the *in vitro* activity of cefiderocol and the four BL/BLI combinations against isolates collected as part of the recent 2019/20 multicentre surveillance study of the Paul-Ehrlich-Society for Infection Therapy (PEG) in Germany. A secondary aim was to compare and evaluate cefiderocol MICs testing methods.

## Materials and methods

### Bacterial isolates

Isolates of different Enterobacterales species and non-fermenters (*n* = 401) were collected at 22 laboratories in Germany between October 2019 and March 2020 for the multicentre surveillance study conducted by the PEG. Two sets of isolates were selected: random samples (set I) and challenge organisms (set II). To verify a consistent distribution within set I isolates, every 13th sample from each laboratory was selected for this study.

#### Random sample of clinical isolates (set I)

Set I (*n* = 199) comprised a random sample of respiratory tract (*n* = 124) and blood culture isolates (*n* = 75). The Enterobacterales group of this set included *Escherichia coli* (*n* = 50), *Klebsiella pneumoniae* (*n* = 33) and *Enterobacter cloacae* complex (*n* = 12). The non-fermenters included *P. aeruginosa* (*n* = 66), *A. baumannii* (*n* = 6) and *Stenotrophomonas maltophilia* (*n* = 32). Within this set of randomly selected isolates, there were seven *E. coli* isolates and five *K. pneumoniae* isolates that encoded CTX-M-type ESBLs. One of these *K. pneumoniae* isolates also carried KPC-2 and was resistant against carbapenems. One *A. baumannii* isolate was carbapenem-resistant due to the presence of OXA-23.

#### Challenge organisms (set II)

Set II (*n* = 202) included multidrug-resistant isolates of *E. coli* (*n* = 65), *K. pneumoniae* (*n* = 44), *E. cloacae* complex (*n* = 19), *P. aeruginosa* (*n* = 66), *A. baumannii* (*n* = 8). Selection criteria were as follows: meropenem-resistance in *A. baumannii* and *P. aeruginosa* (MIC > 8 mg/L), ertapenem resistance (MIC > 0.5 mg/L) and/or meropenem MIC > 0.12 mg/L in *E. coli*, *K. pneumoniae* and *E. cloacae* complex, colistin-resistant isolates (i.e. Enterobacterales, *A. baumannii* and *S. maltophilia* MIC > 2 mg/L; *P. aeruginosa* MIC > 4 mg/L) and ESBL-producing isolates of *E. coli* and *K. pneumoniae*. All *E. coli* isolates, and the majority of *K. pneumoniae* isolates (*n* = 42) were resistant to ceftazidime and/or cefotaxime and carried CTX-M-type and SHV-type ESBL (e.g. CTX-M-15, CTX-M-1, CTX-M-27, SHV-12) or AmpC enzymes (CMY-2, DHA-1). In addition, four *K. pneumoniae* isolates harboured carbapenemase genes *bla*_NDM-1_ _+_ _OXA-48_ (*n* = 1) or *bla*_OXA-232_ (*n* = 3). There were eight meropenem-resistant *A. baumannii* that produced OXA-23 carbapenemase, while two of them harboured an additional ESBL gene (*bla*_PER-1_). Furthermore, five carbapenem-resistant *P. aeruginosa* isolates harboured MBL genes VIM-2 (*n* = 3), GIM-1 (*n* = 1) and IMP-1 (*n* = 1).

### Species identification

Species identification was initially performed by each study site as part of the surveillance study of the PEG. Isolates that were initially identified by Vitek-2 or revealed unusual susceptibility testing results were further analysed using MALDI-TOF mass spectrometry (VITEK-MS, BioMérieux GmbH, Nürtingen, Germany) to verify species identity.

### Antimicrobial susceptibility testing

The following antimicrobial agents were tested with the noted ranges: ceftazidime/avibactam (0.25/4–16/4 mg/L), ceftolozane/tazobactam (0.25/4–8/4 mg/L), imipenem/relebactam (0.06/4–8/4 mg/L), meropenem/vaborbactam (0.06/8–16/8 mg/L) and cefiderocol (0.008–128 mg/L). MICs were determined using the broth microdilution procedure.^10^ The susceptibilities of the comparator compounds were analysed using industrially manufactured 96-well plates and CAMHB from ThermoFisher (Waltham, MA, USA). In parallel, cefiderocol MICs were determined using ComASP microdilution plates (Liofilchem S.r.l., Roseto degli Abruzzi, Italy). The MICs were read visually by the same trained senior scientist and, as far as possible, interpreted according to the species-specific clinical breakpoints approved by EUCAST (v 16.0, January 2026).^11^ Reference strains *E. coli* ATCC 25922 and *P. aeruginosa* ATCC 27853 were used for quality control.

In-house verification of the ComASP cefiderocol test kit was performed by testing triplicates of each reference strain and triplicates of 10 clinical isolates on three different days. Clinical isolates included *E. coli* (*n* = 2), *K. pneumoniae* (*n* = 2), *P. aeruginosa* (*n* = 2), *A. baumannii* (*n* = 2) and *S. maltophilia* (*n* = 2). Testing was always performed by the same technician.

The accuracy of the ComASP test kit was pretested by comparing the MICs of 140 clinical isolates and two reference strains obtained from the ComASP plates (0.008–128 mg/L) with the provided broth to MICs obtained from freshly prepared in-house microtiter plates (0.03–32 mg/L) and iron-depleted CAMHB (ID-CAMHB) prepared according to the procedure described by Hackel *et al*.^12^ The Mueller–Hinton broth used for the medium was purchased from Becton Dickinson (BD BBL; Le Pont de Claix, France). Iron concentration was measured by colorimetric assay before and after depletion (Iron CHEMets^®^ Kit, Aqua Phoenix Scientific, USA). Comparable to the verification, testing was always performed by the same technician. Skipped wells were interpreted according to EUCAST guidelines.

The 140 clinical isolates comprised of *E. coli* (*n* = 43), *K. pneumoniae* (*n* = 27), *E. cloacae* complex (*n* = 13), *A. baumannii* (*n* = 4), *P. aeruginosa* (*n* = 45) and *S. maltophilia* (*n* = 8). The results of this pretest were then evaluated in terms of essential agreement (EA) and bias. The bias was calculated according to ISO 20776–2, including all isolates of the pretest.^13^ In addition, as there were several off-scale MICs (*n* = 26), bias was also calculated using on-scale MICs only.

### Molecular analysis of resistant isolates

Resistance genes were identified by PCR and/or whole-genome sequence analysis (short read sequencing, Illumina) performed for carbapenem-resistant and third-generation cephalosporin-resistant *E. coli* and *K. pneumoniae* isolates, as well as carbapenem-resistant *P. aeruginosa* and *A. baumannii* isolates at the Robert Koch-Institute, Wernigerode Branch, Germany, the National Reference Laboratory for Multidrug-Resistant Gram-negative Bacteria in Bochum, Germany and the Institute for Medical Microbiology, Immunology and Hygiene of the University Hospital of Cologne, Germany.

## Results

### Comparison of the ComASP MIC results with the reference method

Verification of the ComASP test kit performance revealed similar results for the reference strains and the clinical isolates. The majority of MIC values of the reference strains corresponded to the target values (0.125–0.25 mg/L). On two testing days, one reference strain each revealed slightly higher cefiderocol MICs, which were still within the acceptable range (0.5 mg/L). MIC values of the clinical isolates for all triplicates revealed normal day-to-day deviations of ≤ 1 dilution-step compared to the results of the previous in-house BMD testing and comparing the different inocula of each isolate with each other. Results of the pretest are provided in the Supplementary material (available as Supplementary data at *JAC-AMR* Online). This test was originally conducted for 120 clinical isolates only. After the first analysis revealed good correlation, the remaining 281 isolates were tested with ComASP plates only. However, to ensure that all isolates with MICs near and above the Enterobacterales and *P. aeruginosa* resistant breakpoint of 2 mg/L (i.e. MIC values between 1 and 128 mg/L) were included in the final analysis, those isolates were re-tested using both plates in parallel. This resulted in a final number of 140 isolates that were included in the final pretest results. The results of MIC value comparison are shown in Figure S1. The pretest confirmed good correlation of ComASP to the reference method with an overall EA of 89.5% (Table S1) and a bias of +17.5% using all isolates or +2.5% using on-scale MICs only (Figure S2). The lowest EAs by species were 33.3% for *A. baumannii* and 76.9% for *E. cloacae* complex. The EA of all other species was > 90%, with 91.2% for both *E. coli* and *P. aeruginosa,* 95.5% for *K. pneumoniae* and 100.0% for *S. maltophilia*. It should be considered that a limitation of the EA calculation in this study is the small number of isolates included for *A. baumannii*, *S. maltophilia* and *E. cloacae* complex isolates (*n* < 20, respectively).

### Random set of clinical isolates (set I)

Cefiderocol MIC distributions are presented in Table 1. Cefiderocol at a concentration of ≤ 1 mg/L was able to inhibit 100% of the tested isolates of set I. Hence, all Enterobacterales and *P. aeruginosa* isolates would be classified as susceptible according to their EUCAST susceptibility breakpoints. For *A. baumannii* and *S. maltophilia*, cefiderocol breakpoints have not been established due to insufficient clinical data. However, since the *in vitro* activity against both species is comparable to the activity against Enterobacterales, EUCAST has provided evaluation criteria to distinguish between susceptible and potentially resistant isolates (footnote Table 1).^11^

**Table 1.** Distribution of cefiderocol MICs against Gram-negative isolates using ComASP plates

Species	*n*	Numbers of isolates at given MIC (mg/L)												
		≤ 0.03	0.06	0.12	0.25	0.5	1	2	4	8	16	32	64	≥ 128
**Random sample of isolates (set I, *n*** **=** **199)**														
*E. coli*	50	1	5	16	16	10	2							
*K. pneumoniae*	33	2	7	11	10	3								
*E. cloacae* complex	12			1	4	7								
*P. aeruginosa*	66	6	8	25	18	6	3							
*A. baumannii*^b^	6		1	3	2									
*S. maltophilia*^b^	32		3	18	8	2	1							
Subtotal	199	9	24	74	58	28	6							
**Sample of challenge isolates (set II, *n*** **=** **202)**^a^														
*E. coli*	65			5	23	19	13	2	2	1				
*K. pneumoniae*	44		3	2	13	13	9	3	1					
*E. cloacae* complex	19		1	1	3	8	4	2						
*P. aeruginosa*	66		3	17	26	12	6	1	1					
*A. baumannii*^b^	8			1	4		1							*2*
Subtotal	202		7	26	69	52	33	8	4	1				2
**Total**	**401**	**9**	**31**	**100**	**127**	**80**	**39**	**8**	**4**	**1**				**2**

^a^Set II comprised ESBL producers, carbapenemase screen-positive isolates and/or colistin-resistant isolates.

The vertical solid line indicates the EUCAST breakpoints for Enterobacterales and *P. aeruginosa* (S ≤ 2 mg/L, R > 2 mg/L).

^b^According to the EUCAST breakpoint table (v 16.0) instead of a clinical breakpoint an evaluation is provided: ‘Isolates with MIC values ≤ 0.5 mg/L are mostly devoid of resistance mechanisms. Isolates with MICs 1–2 mg/L have acquired resistance mechanisms which may result in impaired clinical response. Isolates with MIC values > 2 mg/L will likely be resistant’.^11^

The MIC values inhibiting 50% and 90% of the isolates as well as the number and percentage of susceptible and resistant isolates according to the available EUCAST breakpoints are presented in Table 2. All 95 Enterobacterales isolates were susceptible to cefiderocol, as well as all comparator agents except ceftolozane/tazobactam, for which two resistant isolates were detected. The MIC50 value of cefiderocol was 0.25 mg/L, which was comparable to the MIC50 values of ceftolozane/tazobactam and ceftazidime/avibactam (≤ 0.25 mg/L each).

**Table 2.** *In vitro* activity of cefiderocol and four newer BL/BLI combinations against Gram-negative isolates stratified by set of isolates (total *n* = 401)

Random sample of isolates (set I, *n* = 199)					Sample of challenge isolates (set II, *n* = 202)^a^				
Antibacterial agent	MIC50 (mg/L)	MIC90 (mg/L)	Number (%) of isolates		Antibacterial agent	MIC50 (mg/L)	MIC90 (mg/L)	Number (%) of isolates	
			S	R				S	R
**Enterobacterales (*n*** **=** **95)**^b^					**Enterobacterales (*n*** **=** **128)**^c^				
FDC (ComASP)	0.25	0.5	95 (100.0)	0 (0.0)	FDC (ComASP)	0.5	1	124 (96.9)	4 (3.1)
FDC (reference BMD	—	—	36 (100.0)	0 (0.0)	FDC (reference BMD)	—	—	43 (91.5)	4 (8.5)
CTT	≤ 0.25	0.5	93 (97.9)	2 (2.1)	CTT	0.5	8	109 (85.2)	19 (14.8)
CTV	≤ 0.25	0.5	95 (100.0)	0 (0.0)	CTV	≤ 0.25	1	127 (99.2)	1 (0.8)
IMR	0.12	0.25	95 (100.0)	0 (0.0)	IMR	0.12	0.25	124 (96.9)	4 (3.1)
MEV	≤ 0.06	≤ 0.06	95 (100.0)	0 (0.0)	MEV	≤ 0.06	≤ 0.06	124 (96.9)	4 (3.1)
***P. aeruginosa* (*n*** **=** **66)**					***P. aeruginosa* (*n*** **=** **66)**				
FDC (ComASP)	0.12	0.25	66 (100.0)	0 (0.0)	FDC (ComAsp)	0.25	1	65 (98.5)	1 (1.5)
FDC (reference BMD)	—	—	19 (100.0)	0 (0.0)	FDC (reference BMD)	—	—	23 (100.0)	0 (0.0)
CTT	1	4	63 (95.5)	3 (4.5)	CTT	1	≥ 16	53 (80.3)	13 (19.7)
CTV	2	8	64 (97.0)	2 (3.0)	CTV	4	≥ 32	45 (68.2)	21 (31.8)
IMR	0.5	2	64 (97.0)	2 (3.0)	IMR	2	≥ 16	51 (77.3)	15 (22.7)
MEV	0.5	4	63 (95.5)	3 (4.5)	MEV	8	≥ 32	39 (59.1)	27 (40.9)
***A. baumannii* (*n*** **=** **6)**					***A. baumannii* (*n*** **=** **8)**				
FDC (ComASP)	0.12	0.25	No EUCAST breakpoints		FDC (ComASP)	0.25	≥ 256	No EUCAST breakpoints	
CTT	0.5	≥ 16			CTT	≥ 16	≥ 16		
CTV	4	≥ 32			CTV	≥ 32	≥ 32		
IMR	0.25	≥ 16			IMR	≥ 16	≥ 16		
MEV	0.25	8			MEV	≥ 32	≥ 32		
***S. maltophilia* (*n*** **=** **32)**									
FDC (ComASP)	0.12	0.25	No EUCAST breakpoints						
CTT	≥ 16	≥ 16							
CTV	16	≥ 32							
IMR	≥ 16	≥ 16							
MEV	≥ 32	≥ 32							

^a^See footnote of Table 1 for details.

^b^ *Enterobacter cloacae* complex (*n* = 12), *Escherichia coli* (*n* = 50) and *Klebsiella pneumoniae* (*n* = 33).

^c^ *Enterobacter cloacae* complex (*n* = 19), *Escherichia coli* (*n* = 65) and *Klebsiella pneumoniae* (*n* = 44).

Abbreviations: FDC, cefiderocol; CTT, ceftolozane/tazobactam; CTV, ceftazidime/avibactam; IMR, imipenem/relebactam; MEV, meropenem/vaborbactam.

While cefiderocol inhibited all *P. aeruginosa* isolates of set I, the most effective comparator agents were ceftazidime/avibactam and imipenem/relebactam with a susceptible rate of 97% (64/66) each (Table 2). Among the *A. baumannii* isolates, all were inhibited by cefiderocol at ≤ 0.25 mg/L, while more than 50% of all isolates (4/6) were inhibited at ≤ 0.12 mg/L (Tables 1 and 2). Cefiderocol MICs of the *S. maltophilia* isolates ranged from ≤ 0.03 to 1 mg/L with MIC50 and MIC90 values of 0.12 and 0.25 mg/L (Tables 1 and 2). Interestingly, all four comparator agents were less active against *S. maltophilia* with MIC50 and MIC90 values of ≥ 16 mg/L.

### Challenge isolates (set II)

According to ComASP plates, there were seven isolates of set II that displayed cefiderocol MICs > 2 mg/L (Table 1). Two *E. coli* isolates (encoding CTX-M-15 +/− AmpC-type CMY-42), one *K. pneumoniae* (encoding CTX-M-15, NDM-1 and OXA-48) and one *P. aeruginosa* isolate revealed MICs of 4 mg/L, while one-third *E. coli* isolate (encoding CTX-M-15) displayed a MIC of 8 mg/L. The *P. aeruginosa* isolate was not molecularly analysed prior to the study, as it was susceptible to carbapenems. The highest cefiderocol MICs were observed for two *A. baumannii* isolates with 128 and ≥ 256 mg/L, respectively (both encoding OXA-23 and PER-1).

Overall, cefiderocol susceptibility of Enterobacterales isolates in set II was 96.9% (124/128), with MIC50 and MIC90 values of 0.5 and 1 mg/L (Tables 1 and 2). Among the comparator agents, ceftazidime/avibactam displayed the highest *in vitro* activity with a resistance rate of 0.8% (1/128) and very low MIC50 and MIC90 values of ≤ 0.25 and 1 mg/L, respectively (Table 2). The highest resistance rate was found for ceftolozane/tazobactam with 14.8% (19/128). In *P. aeruginosa*, cefiderocol showed the highest activity of all tested agents (resistance rate 1.5% (1/66); Tables 1 and 2), followed by ceftolozane/tazobactam with a resistance rate of 19.7% (13/66). Regarding *A. baumannii* isolates cefiderocol was found to have the lowest MIC50 value with 0.25 mg/L followed by ≥ 16 mg/L for ceftolozane/tazobactam and imipenem/relebactam, as well as ≥ 32 mg/L for ceftazidime/avibactam and meropenem/vaborbactam (Table 2).

Interestingly, using reference BMD cefiderocol plates, there were minor changes in the interpretation of the results in set II isolates: Two cefiderocol-resistant isolates (*P. aeruginosa* and *E. coli*) showed one or two dilution step lower MICs with the in-house plates compared to ComASP and would therefore be interpreted as susceptible. Another *E. coli* isolate revealed a one dilution step higher MIC with the in-house plate and would therefore be interpreted as resistant. Taken together, the differences in the two plates would lead to a lower resistance rate in set II *P. aeruginosa* isolates. Interpretation of the Enterobacterales isolates would remain the same.

### Resistant isolates (set I and II)

MIC distributions in all sequenced ESBL and carbapenemase producers, as well as colistin-resistant isolates of set I and II, are displayed together in Table 3. ESBL and carbapenemase genes were detected in 109 and 19 isolates, respectively. Colistin resistance was confirmed in 24 isolates (Table 3). Overall, cefiderocol at ≤ 2 mg/L inhibited 96.3% (105/109) of ESBL producers, 84.2% (16/19) of carbapenemase-producers and 95.8% (23/24) of the colistin-resistant isolates. The comparator agents showed varying *in vitro* activity compared to cefiderocol in this subset of isolates (Table 3).

**Table 3:** **Distribution of cefiderocol MICs and comparator agents against resistant subgroups of Gram-negative isolates (set I plus set II; *n*** **=** **152)**

Bacterial group	Numbers of isolates at given MIC (mg/L)												
	≤ 0.03	0.06	0.12	0.25	0.5	1	2	4	8	16	32	64	≥ 128
**ESBL-producing (*n*** **=** **109)**^a^													
FDC		2	8	39	32	20	4	3	1				
CTT				**29**	44	16	9	2	4	*5*			
CTV				**89**	11	5	1	1	1		*1*		
IMR		**3**	73	26	3			1	2	*1*			
MEV		**103**	1		1						*4*		
**Carbapenemase-producing (*n*** **=** **19)**^b^													
FDC		2	2	7	4	1		1					*2*
CTT									2	*17*			
CTV				**1**		3				2	*13*		
IMR					1			1	2	*15*			
MEV		**1**							1		*17*		
**Colistin-resistant (*n*** **=** **24)**^c^													
FDC		1	4	4	7	4	3	1					
CTT				**2**	11	5	1	3	1	*1*			
CTV				**12**	4	1	5			1	*1*		
IMR			7	10	4	1		1		*1*			
MEV		**17**		2	1	1		1		1	*1*		

^a^ *E. coli* (CTX-M group 1, *n* = 45; CTX-M group 9, *n* = 23; CTX-M group 1 + CTX-M group 9, *n* = 1; SHV-12, *n* = 1) and *K. pneumoniae* (CTX-M group 1, *n* = 35; CTX-M group 9, *n* = 4).

^b^ *A. baumannii* (OXA-23, *n* = 7; OXA-23(+PER-1), *n* = 2), *K. pneumoniae* (OXA-232, *n* = 3; KPC-2, *n* = 1; NDM-1 + OXA-48, *n* = 1) and *P. aeruginosa* (IMP-1, *n* = 1; GIM-1, *n* = 1; VIM-2, *n* = 3).

^c^ *E. cloacae* (*n* = 13), *E. coli* (*n* = 1); *K. pneumoniae* (*n* = 4) and *P. aeruginosa* (*n* = 6). Abbreviations: FDC, cefiderocol; CTT, ceftolozane/tazobactam; CTV, ceftazidime/avibactam; IMR, imipenem/relebactam; MEV, meropenem/vaborbactam. Numbers in bold include isolates with MIC < value shown; numbers in italic include isolates with MIC > the highest concentration tested.

The cefiderocol distribution according to species and type of ESBL or carbapenemase is shown in Table 4. As stated above, cefiderocol MIC values > 2 mg/L were associated with OXA-23 + PER-1-producing *A. baumannii,* CTX-M-15-encoding *E. coli* and NDM-1 + OXA-48 + CTX-M-15-producing *K. pneumoniae*. None of the MBL-producing *P. aeruginosa* isolates was resistant to cefiderocol.

**Table 4.** Distribution of cefiderocol MICs in isolates with characterized ESBL/carbapenemase genes (*n* = 128)

Species	Ambler class	Type of ESBL + carbapenemase	MIC (mg/L)												
			≤0.03	0.06	0.12	0.25	0.5	1	2	4	8	16	32	64	≥128
*E. coli*	A	CTX-M group 1 (*n* = 45)			3	12	15	10	2	2	1				
		CTX-M group 9 (*n* = 23)			3	10	7	3							
		CTX-M group 1 + CTX-M group 9						1							
		SHV-12				1									
*K. pneumoniae*	A	CTX-M group 1 (*n* = 30)	1	1	14	6	6	2							
	A (+ B + D)	CTX-M group 1 + OXA-232 (*n* = 3)				3									
		CTX-M group 1 + KPC-2			1										
		CTX-M group 1 + NDM-1 + OXA-48							1						
	A	CTX-M group 9 (*n* = 4)	1	1	1	1									
Total (*n* = 109)				2	8	39	32	20	4	3	1				
**Species**	**Ambler class**	**Type of carbapenemase + ESBL**	**MIC (mg/L)**												
			**≤0.03**	**0.06**	**0.12**	**0.25**	**0.5**	**1**	**2**	**4**	**8**	**16**	**32**	**64**	**≥128**
*A. baumannii*	D (+ A)	OXA-23 + PER-1 (*n* = 2)													2
	D	OXA-23 (*n* = 7)			1	5		1							
*K. pneumoniae*	D (+ A)	OXA-232 + CTX-M group 1 (*n* = 3)					3								
	A	KPC-2 + CTX-M group 1				1									
	B + D (+ A)	NDM-1 + OXA-48 + CTX-M group 1								1					
*P. aeruginosa*	B	GIM-1				1									
		IMP-1					1								
		VIM-2 (*n* = 3)		2	1										
Total (*n* = 19)				2	2	7	4	1		1					2

## Discussion

AMR has been described as one of the biggest threats to global health.^14^ Besides the possibility of untreatable bacterial infections and hence increased mortality, healthcare systems would also suffer from increased costs due to drug consumption and hospital admissions.^15^

Currently, carbapenemase-producing Enterobacterales play a minor clinical role in Germany. According to the ECDC, the rate of carbapenem-resistant isolates was < 0.1% for *E. coli* and 0.9% for *K. pneumoniae* in 2019, the time period when the isolates for this present study were collected.^16^ In contrast, carbapenem resistance in *A. baumannii* and *P. aeruginosa* is a reason for concern in Germany. In 2019, the rate of carbapenem-resistant *P. aeruginosa* was 12.9%, while 2.2% of *Acinetobacter spp.* were carbapenem resistant.^16^ Cefiderocol and the comparator agents investigated in this study are considered to be promising candidates for the treatment of some of these pathogens.^7^ However, resistance to BL/BLI combinations is emerging partly due to the high degree of chemical similarity of the different components.^17^

In this study, we compared the ComASP cefiderocol MIC test kit to the reference method of broth microdilution using freshly prepared in-house plates (Figure S1, Table S1 and Figure S2). Cefiderocol susceptibility testing is often considered challenging due to the unique mode of action of the compound that requires iron-depleted broth to be tested in broth microdilution.^18,19^ In this study, however, we have found good correlation between both methods with a species-dependent EA of 89.5% (Table S1) and an overall bias of +17.5% (calculated according to ISO, including all isolates of the pretest (*n* = 140)) or +2.5% (calculated with on-scale MICs only (*n* = 114); see Figure S2 for more information). Skipped wells were determined for 4% (16/401) of all tested isolates.

The EA value is comparable to the one determined by Bianco *et al*.^20^ which was 84% for the same species that were tested here. Another study by Koeth *et al*.^21^ has compared the ComASP test kit to the BMD reference method and received an EA between 82.7% (CLSI read, i.e. ignoring faint growth) and 95.3% (ComASP read, i.e. 100% inhibition), highlighting the importance of the reading method. In the present study, we analsed MIC values according to the EUCAST reading guide which is comparable to CLSI: ‘The MIC is read as the first well in which the reduction of growth corresponds to a button of < 1 mm or is replaced by the presence of light haze/faint turbidity (≥ 80% reduction in haze as compared to the growth control)’ (Reading guide EUCAST BMD). In the presented study, cefiderocol showed potent activity against the included isolates with inhibition rates of 100% (set I; MIC50/90: 0.12/0.5 mg/L) and 97.5% (set II; MIC50/90: 0.25/1 mg/L) at a concentration of ≤ 2 mg/L (Tables 1 and 2). This is comparable to the two previous studies that were conducted in a similar design.^5,9^ In the Enterobacterales group of both sets, only ceftolozane/tazobactam displayed noticeably lower activity against the isolates than the other comparator agents, which all showed a comparable susceptibility rate of > 95% (Table 2). Cefiderocol resistance of one single *K. pneumoniae* isolate in set II was associated with the presence of MBL gene *bla*_NDM-1_ (Tables 3 and 4). As this MBL has been described to be associated with cefiderocol resistance,^22^ it is likely to be an important factor in the respective isolate, while probably not the sole resistance mechanism as cefiderocol resistance is considered to be multifactorial. In addition, there were three cefiderocol-resistant *E. coli* isolates with CTX-M-type ESBL production only. Cefiderocol resistance in these isolates might be associated with alterations in the iron uptake system as described by Price *et al*.,^23^ which needs further investigation.

In *P. aeruginosa,* meropenem/vaborbactam and ceftazidime/avibactam were the least active agents tested (Table 2). In contrast, cefiderocol was the most potent substance with susceptibility rates of 100% (set I) and 98.5% (set II) and the only agent that reached a susceptibility rate of > 90% in the *P. aeruginosa* isolates of set II (Tables 1 and 2). A similar observation was made by Gill *et al*., who observed an overall cefiderocol susceptibility of 95% in a global collection of carbapenem-resistant *P. aeruginosa*.^24^ Cefiderocol is also actively transported into *P. aeruginosa* cells through the iron transport system,^6^ which is often seen as the reason for its high activity against this species.

Cefiderocol displayed potent activity against *A. baumannii* (*n* = 14) isolates included in this study with the majority of isolates (12/14) displaying MIC values of < 2 mg/L (Tables 1 and 2). Interestingly, Seifert *et al*.^25^ have reported a similar activity of cefiderocol using a global collection of carbapenem-resistant *A. baumannii* with MIC50/90 values of 0.5 mg/L and 4 mg/L, respectively. In this international study, all NDM-producing (*n* = 6) and IMP-producing (*n* = 1) isolates were resistant to cefiderocol.

The two *A. baumannii* isolates of the present study, which produced OXA-23 and an additional PER-1 ESBL, showed high cefiderocol MIC values of ≥ 128 mg/L (Tables 3 and 4). PER-1 is sometimes referred to as the key resistance mechanism behind *A. baumannii* cefiderocol resistance, while in other publications, it is only stated as one component of a multifactorial resistance mechanism.^26,27^ The catalytic efficiency of PER-1 for cefiderocol has been demonstrated previously by enzyme kinetics.^26^ Interestingly, recombinant *bla*_PER-1_ expressing *E. coli* showed a 64-fold increase in cefiderocol MIC.^27^ However, the recombinant strain was still susceptible, leading to the suggestion that there are other factors that support PER-1-driven hydrolysis of cefiderocol in *A. baumannii* which may involve reduced influx of the drug, similar to what is known for carbapenems.

In *S. maltophilia*, cefiderocol had the lowest MIC50 and MIC90 values of all comparators (Tables 1 and 2). This has also been observed in other studies, such as Delgado-Valverde *et al*.^28^ and Biagi *et al*.^29^ Cefiderocol has also been shown to be highly active in treating experimental *S. maltophilia* pneumonia.^30^

In conclusion, our results correlated with results of the previous study using isolates collected between 2016 and 2017. NDM-1 and PER-1 were found to be associated with cefiderocol MICs > 2 mg/L in *K. pneumoniae* and *A. baumannii*, respectively. Cefiderocol resistance in *E. coli* could be due to alterations in the iron uptake system, which needs further investigation. In comparison to the BL/BLI combinations, cefiderocol showed the most potent activity against *P. aeruginosa*, *A. baumannii* and *S. maltophilia*. This highlights the importance of parallel testing of all last resort drugs in order to find the most promising therapy for critically ill patients.

## Supplementary Material

dlag103_Supplementary_Data

## Supplementary data

Figures S1 to S2 and Tables S1 are available as Supplementary data at *JAC-AMR* Online.
