# Supplementary material for: Update on the comparative in vitro activity of cefiderocol and four β-lactam–β-lactamase-inhibitor combinations against clinically important Gram-negative pathogens
Source: JAC Antimicrob Resist. 2026 Jun 8;8(3):dlag103. doi: 10.1093/jacamr/dlag103 (PMC13243917; doi:10.1093/jacamr/dlag103)
Supplement: dlag103_Supplementary_Data [file dlag103_supplementary_data.docx]

**Supplementary material**

Figure S1: Scattergram analysis of cefiderocol MICs for a subset of Gram-negative clinical isolates (n = 140) – performance of BMD reference method versus ComASP plates

Table S1: Distribution of cefiderocol log2 dilution step differences and essential agreement between MIC resuts of ComASP plates compared to BMD reference method

1. **Bias calculated according to ISO 20776:2-2021**

- All isolates of the pre-test are included (n=140)
- Isolates greater than the reference method:

1. Range < 0.03 – 32 with MICs greater than the reference method = 23 + 8 + 9 + 8 + 3 + 3 +1 + 1 + 1 = 57

2. Range < 0.03 – 32, total number of MICs = 140 - 1 = 139

3. Percentage: 100 * (57/139) = 41.0%

- Isolates less than the reference method:

1. Range 0.06 – >32 with MICs less than the reference method = 0 + 0 + 8 + 5 + 6 + 7 + 1 + 0 = 27

2. Range 0.06 – >32, total number of MICs = 140 - 25 = 115

3. Percentage: 100 * (27/115) = 23.5%

- Difference between percentage of results greater than the reference method and less than the reference method (**bias**):
  41.0% - 23.5% = **17.5%** (acceptable as it is within the range of -30.0-+30.0%)

1. **Bias calculated using on-scale MICs only (n=114)**

- MICs > the highest concentration tested (> 64 mg/L) and ≤ the lowest concentration tested (≤ 0.03 mg/L) are excluded, due to the fact that it is not possible to determine whether MICs of the test method are lower or higher compared to the reference method.
- Isolates greater than the reference method:

1. 0.06 – 16 with MICs greater than the reference method = 8 + 9 + 8 + 3 + 3 + 1 + 1 = 33

2. 0.06 – 16, total number of MICs = 114 - 1 = 113

3. Percentage: 100 * (33/113) = 29.2%

- Isolates less than reference method:

1. 0.12 – 32 with MICs less than the reference method = 0 + 8 + 5 + 6 + 7 + 1 + 1 = 28

2. 0.12 – 32, total number of MICs = 114 - 9 = 105

3. Percentage: 100 * (28/105) = 26.7%

- Difference between percentage of results greater than the reference method and less than the reference method (**bias**):
  29.2% - 26.7% = **2.5%** (acceptable as it is within the range of -30.0-+30.0%)

Figure S2: Detailed calculation of the bias including all isolates (A) and isolates with on-scale MICs only (B)
